# Child Welfare Reform: A Scoping Review

**DOI:** 10.3390/ijerph192114071

**Published:** 2022-10-28

**Authors:** Jill R. McTavish, Christine McKee, Masako Tanaka, Harriet L. MacMillan

**Affiliations:** 1 Offord Centre for Child Studies, Department of Psychiatry and Behavioural Neurosciences, McMaster University, 293 Wellington Street North, Hamilton, ON L8L 8E7, Canada; 2 Department of Pediatrics, McMaster University, 1280 Main Street West, Hamilton, ON L8S 4K1, Canada

**Keywords:** child maltreatment, child welfare, child protection, reform, scoping review, policy

## Abstract

While there have been ongoing calls to reform child welfare so that it better meets children’s and families’ needs, to date there have been no comprehensive summaries of child welfare reform strategies. For this systematic scoping review, we summarized authors’ recommendations for improving child welfare. We conducted a systematic search (2010 to 2021) and included published reviews that addressed authors’ recommendations for improving child welfare for children, youth, and families coming into contact with child welfare in high-income countries. A total of 4758 records was identified by the systematic search, 685 full-text articles were screened for eligibility, and 433 reviews were found to be eligible for this scoping review. Reviews were theoretically divided, with some review authors recommending reform efforts at the macro level (e.g., addressing poverty) and others recommending reform efforts at the practice level (e.g., implementing evidence-based parenting programs). Reform efforts across socioecological levels were summarized in this scoping review. An important next step is to formulate what policy solutions are likely to lead to the greatest improvement in safety and well-being for children and families involved in child welfare.

## 1. Introduction

Child maltreatment includes physical, sexual, and emotional abuse, neglect, and children’s exposure to intimate partner violence [1,2]. It is a prevalent experience with many potentially serious negative consequences, such as injuries, developmental delay, anxiety and mood disorder symptoms, poor peer relationships, substance use and other risky behaviours [3,4,5,6,7]. Gilbert and colleagues [8] have specified two approaches to the response of child maltreatment: a child safety approach, where government-based agencies are responsible almost entirely for the response to child maltreatment; and a child and family welfare approach, where child welfare organizations respond to allegations of child maltreatment alongside referrals regarding other vulnerable children. The former involves a more investigative response whereas the latter involves more of a preventative and service-oriented response. At the time of publication, Gilbert et al. [8] noted that Canada, the United States, and Australia have taken a child safety approach, whereas New Zealand, the United Kingdom, and several western European countries have taken a child and family welfare approach.

In this article we refer to child welfare as a set of government and private services primarily designed to protect children from child maltreatment, encourage family stability, and, when necessary, arrange foster care and adoptions and child protection services (CPS) as a narrower set of services within child welfare that investigate allegations of child maltreatment [9]. Data from several countries suggests a high cumulative number of children are being investigated for experiences of maltreatment—in New Zealand approximately 25% of children were referred to CPS by age 18 [10] and in the United States 37.4% were referred by age 18 [11]. Certain groups are more likely to come into contact with CPS than others related to a complex array of factors, including colonialism, racism, discrimination, and poverty [12,13]. For example, data from the 2019 First Nations Canadian Incidence Study of Reported Child Abuse and Neglect (CIS) reported on child welfare investigations involving First Nations and non-Indigenous children. This study found that, in Canada, First Nations youth are 3 to 4 times more likely to be the focus of a child protection report and 17 times more likely to experience an out-of-home placement related to a CPS investigation [12]. 

In general, the evidence regarding the effects of child welfare contact on children is absent or mixed [14,15]. It is challenging to assess the benefits and harms of child welfare interventions, including out-of-home care, as it is unclear if the differences between groups are reflective of the services or differences in a broad range of baseline factors, including socioeconomic status, caregiver educational status, immigration status, family risks, child welfare worker propensity to place children, and children’s safety and well-being at the time of placement [16,17,18,19,20,21]. There has been increasing recognition of the high service demands on CPS and child welfare more broadly in high-income countries [22], as well as ongoing calls to reform child welfare so that it better meets children’s and families’ needs [23,24,25,26]. While much attention has been paid to the need for primary prevention of child maltreatment [27,28,29,30,31,32,33,34,35,36,37], to date there have been no comprehensive summaries of authors’ recommendations for improving child welfare (secondary and tertiary prevention efforts involving child welfare).

This scoping review of reviews summarized authors’ recommendations for improving child welfare. This review was guided by the following research question: What are authors’ recommendations for improving child welfare across socioecological levels, including at the societal-level (e.g., policy), community-level (e.g., coordination of services), institutional level (e.g., child welfare initiatives), relationship level (e.g., ideal strategies to support children and families), and individual level (e.g., training)? A summary of author-proposed child welfare solutions across socioecological levels is important for policy and practice efforts to improve child welfare responses, including informing next steps for child welfare reform.

## 2. Materials and Methods

The present review followed principles of a systematic scoping review. According to Daudt et al. [38], a scoping review is a type of research synthesis that aims to “map the literature on a particular topic or research area and provides an opportunity to identify key concepts; gaps in the research; and types and sources of evidence to inform practice, policymaking, and research” (p. 8). While systematic reviews tend to have clear a priori inclusion and exclusion criteria, scoping reviews are guided by broader research questions related to mapping the literature base [39]. Unlike systematic reviews, scoping reviews also do not evaluate the quality of included studies. Most scoping reviews do not aim to synthesize the existing evidence base, but to map or chart the data within the evidence base. Systematic scoping reviews, alternatively, blend characteristics of systematic reviews and scoping reviews and require the following: an a priori protocol, descriptions about the objectives of the review, inclusion and exclusion criteria (at least the population, concept, and context), and methods for conducting the review [39]. Below we detail the proposed methods for the present systematic scoping review. An earlier version of this review was funded in a project to investigate child welfare reform in Canada, which led to the focus on high-income countries.

### 2.1. Inclusion Criteria

Systematic scoping reviews typically specify included populations, as well as the focusing ‘concept’ and ‘context’ of the review (see Table 1). In this scoping review we included reviews that addressed authors’ recommendations for improving child welfare for children, youth, and families coming into contact with child welfare in high-income countries. Reviews that focused on child maltreatment only (e.g., etiology, risk and protective factors, or health outcomes of child maltreatment) without discussing aspects of child welfare reform were not included.

### 2.2. Systematic Search

The systematic search was conducted by an information professional (JRM). Index terms and keywords related to child welfare and out-of-home care (e.g., foster care, out-of-home care, child protection investigation) and reviews (e.g., scoping review, meta-analyses) were used in the following databases: Medline (OVID), PsycINFO, Sociological Abstracts, and Social Science Citation Index (see Table 2 for an example search strategy). Databases were searched for results from the past 10 years (1 January 2010 to 4 June 2021, when the search was conducted). Titles and abstracts and full-text articles were screened by one author (JRM) for inclusion and a second author (CM) screened all excluded titles and abstracts and full-text articles to ensure relevant articles were not excluded. Differences in screening decisions were resolved by consensus.

### 2.3. Data Analysis

Data analysis summarized the included reviews with attention to the: number of reviews published; type of review (e.g., meta-analysis, rapid review); populations included in reviews (e.g., children, parents, or families involved in child welfare or out-of-home care, child welfare professionals, foster carers); and thematic focus of the included review (e.g., health sector interventions and outcomes, child welfare sector interventions and outcomes, international focus). Data was primarily drawn from the article title, abstract, study inclusion criteria, and methods. For some narrative reviews, such as those without a methods section, information was drawn from the entire article. Analysis also summarized the socioecological level of authors’ recommendations to improve child welfare (e.g., policy-related versus institutional proposal for reform). The socioecological model is commonly used in violence prevention research to outline the range of factors influencing risk and prevention of violence [40]. The model was used in the present scoping review to organize and analyze trends and gaps in authors’ recommendations for child welfare reform. This information was primarily drawn from the discussion of included articles. For some narrative reviews, such as those without a clear discussion section or those that offered recommendations throughout, information was drawn from the entire article.

Codes for types of reviews reflected review authors’ descriptions of their work; review methods were not scrutinized to determine the accuracy of the authors’ descriptions of their work. For example, if the authors of a review called their work a systematic review in the title or methods, it was labelled as a systematic review even if it did not critically appraise articles. Reviews that did not identify their work as a specific type of review were labelled as narrative reviews. Population data was primarily drawn from the review’s inclusion criteria, if available. One code for the population was preferred, when possible. For example, if the review included children in “out-of-home care” the population was coded as “out-of-home care, children” unless it was obvious that results were only provided for children in a specific type of out-of-home care (e.g., only children in foster care). However, there were many instances where reviews addressed multiple populations, such as children in foster care and foster carers, in which case both populations were coded. 

Coding for the main theme of the review and authors’ recommendations for child welfare reform was primarily inductive and akin to a high-level thematic analysis [41]. Thematic analysis involves familiarizing oneself with the data, generating initial codes, searching for themes, reviewing themes, defining and naming themes, and writing up the themes in a manuscript form. For data analysis, one author (JRM) reviewed all the articles and developed a high-level coding scheme for the thematic focus of articles and authors’ recommendations for child welfare reform. For the thematic focus of the articles, two authors (CM, MT) then used the coding scheme to independently code 10 percent (*n* = 45) of the included articles using the coding scheme. Differences between the primary authors (JRM) and other authors coding (CM, MT) were then discussed, the coding scheme was refined, and used by the primary author to recode all of the articles. 

To analyze the authors’ recommendations for improvement, one author (JRM) coded recommendations for approximately half the included articles (codes were typically found in the discussion, as mentioned above) and developed a coding scheme. Two other authors (CM, MT) coded 10% of additional articles (*n* = 45) using this coding scheme. The coding scheme was then updated by both expanding it to include additional, needed codes (e.g., mental health, intimate partner violence) and collapsing it to merge less-used codes (e.g., “disability” was placed under “complex needs”). Following this, each author (CM, HLM, JRM, MT) coded approximately a quarter of articles with the coding scheme for authors’ recommendations for improvement.

## 3. Results

**Figure 1:**
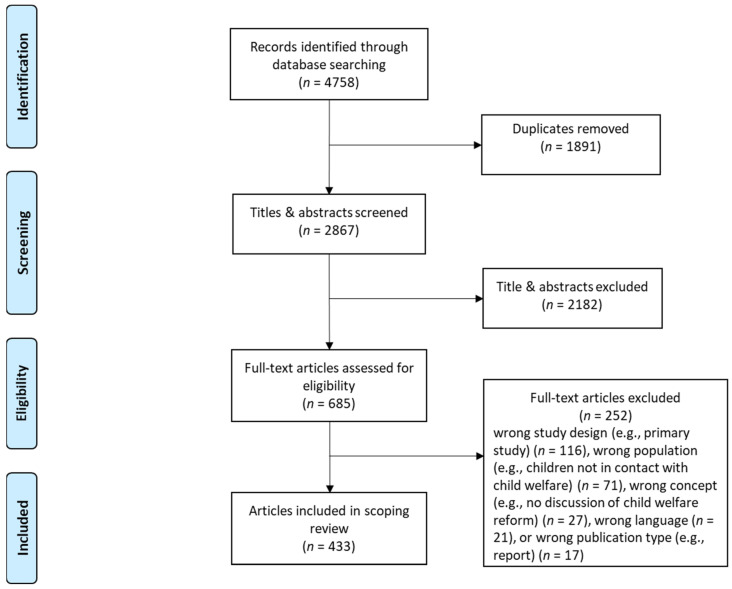
PRISMA flow diagram.

A total of 4758 records was identified in the systematic search, 2867 titles and abstracts were screened, and 685 full-text articles were screened for eligibility. A total of 433 were found to be eligible for this scoping review (see Figure 1).

### 3.1. Types of Reviews

Table 3 indicates the types of reviews addressed by included articles. The majority of reviews were narrative reviews, followed by systematic reviews, scoping reviews, meta-analyses, integrative reviews, rapid reviews, and meta-syntheses. One mapping review was also included. Narrative reviews [42] “generally are comprehensive and cover a wide range of issues within a given topic” (p. 104) and tend to provide an overview of “background knowledge, evolving concepts and controversy” (p. 104) within a field. Systematic reviews [43] generally involve a summary of literature “that uses explicit and reproducible methods to systematically search, critically appraise, and synthesize [literature] on a specific issue” (p. 10). Scoping reviews [38], as discussed above, “map the literature on a particular topic or research area and provide an opportunity to identify key concepts; gaps in the research; and types and sources of evidence to inform practice, policymaking, and research” (p. 8). Meta-analyses [43] involve “the combination of data from several independent primary studies that address the same question to produce a single estimate like the effect of treatment or risk factor” (p. 10). Integrative reviews [44] are a broad type of research review that allow for the “inclusion of experimental and non-experimental research in order to more fully understand a phenomenon of concern” (p. 547). Rapid reviews [45] are “a form of knowledge synthesis in which components of the systematic review process are simplified or omitted to produce information in a timely manner” (p. 2). Meta-syntheses [46] involve “the systematic review and integration of findings from qualitative studies” (p. 1). Mapping reviews [47] are like scoping reviews in that they “map out and categorize existing literature” (p. 94) but their results tend to be more visual compared to scoping reviews.

### 3.2. Population Focus

As is shown in Table 4, the majority of reviews focused on out-of-home care, followed by families (families, parents, children) involved with child welfare. Some articles also focused on child welfare organizations, child welfare professionals, and interdisciplinary initiatives (e.g., children’s advocacy centres or family drug courts).

### 3.3. Thematic Focus

Table 5 summarizes the thematic focus of included articles across socioecological levels [40]. As discussed above, the socioecological model helps to organize core themes in violence prevention, pointing to research trends and gaps. Each of the four levels of the socioecological model is shaded in Table 5 with examples of the level listed (e.g., “Society” themes tend to focus on international and national laws and policies whereas “Individual” themes tend to focus on knowledge, attitudes and skills of individuals). Within each socioecological level of themes, sometimes groups of sub-themes were found. For example, many articles focused on interventions or programs (e.g., parenting programs) and services (e.g., foster care) from different sectors (child welfare, health, education, research, justice, housing). Counts of themes are listed in Table 5; these counts indicate the number of articles that addressed the theme (e.g., in the Excel coding file, 40 articles are coded as discussing the theme “collaboration models, strategies, and components”). Headings associated with socioecological theme levels (e.g., “Society—laws and policy”) and bolded sub-theme levels (e.g., “Child welfare professionals”) in Table 5 have no associated counts as these lines are provided to help visually organize the thematic focus of included articles. Each level is discussed in more detail below. While relationships were a key factor mentioned in many articles, they were the primary focus of very few articles (and so are not coded in Table 5). Additional codes related to relationships are found in the next section (improvements across socioecological levels).

#### 3.3.1. Societal-Level Thematic Focus

Some reviews addressed international or national factors affecting child welfare. These reviews addressed cross-country analyses of specific aspects of child welfare (e.g., development of family support services compared across countries); international influences, such as international actors influencing child welfare (e.g., international governmental organizations); and the impact of human rights treaties. National aspects of child welfare were also discussed, such as aspects of child welfare structure (e.g., history of child welfare, universal services versus targeted services, types of services offered at national level, funding streams, child welfare standards); national child welfare legislation and policies (e.g., mandatory reporting, COVID policies affecting child welfare, austerity policies); systemic disadvantage in child welfare (e.g., poverty, colonialism, disparity); and national actors influencing child welfare (e.g., courts, policymakers, child advocates).

#### 3.3.2. Community-Level Thematic Focus

Some review articles addressed multi- and inter-disciplinary collaboration models, strategies, and components.

#### 3.3.3. Institutional-Level Thematic Focus

It is notable that the majority of reviews addressed interventions, services, programs, and outcomes associated with child welfare or health. Reviews coded for child welfare interventions, services, programs, and outcomes included the following primarily child services and outcomes related to: placement (e.g., placement stability, breakdown, reunification, preventing re-entry); biological family (placement prevention, family functioning, contact, and programs directed at biological family, such as intensive family preservation or parenting programs); service usage (e.g., rates of service usage); participation (e.g., participation of children and families in child welfare services); transition from care (e.g., programs and services related to transitioning from care); safety (safety, recurrence, occurrence, peer violence in out-of-home care); and foster or kinship care as a service (e.g., comparing foster care as a service versus kinship care). Reviews coded for health interventions, services, programs, and outcomes addressed the following child services and outcomes: mental health (e.g., mental health diagnoses and disorders, eating and food-related problems, well-being, resilience); social health (e.g., attachment, peer relationships, identity development, social support); physical health (e.g., development, immunizations, medication management, physical activity, sexual health); and service usage (e.g., rates of service usage). 

Services, programs, interventions, and outcomes related to other sectors were less commonly reviewed, but included those related to education (e.g., speech/language, academic skills, academic achievement), research (e.g., fidelity), justice, (e.g., delinquency, crime prevention, offending behaviour), and housing (e.g., risk for homelessness, housing instability).

#### 3.3.4. Individual-Level Thematic Focus

Some review articles addressed aspects of child welfare professionals and foster or kinship carers. Specifically, they focused on child welfare professionals’ knowledge, skills, abilities, and needs; decision-making; personal characteristics; and the impact of their role (e.g., vicarious trauma). Similarly, for foster and kinship carers, articles discussed personal characteristics and knowledge, skills, abilities, and needs.

### 3.4. Improvements across Socioecological Levels 

Table 6 summarizes author-proposed improvements for child welfare across socioecological levels. Each level is discussed below. Given the number of articles addressing each theme, only a small sample of citations are listed after each theme below. 

#### 3.4.1. Societal-Level Suggestions for Improvements

Some review authors noted the need for better funding and political support for child welfare [48,49,50], including resources for supporting kinship families [51,52]. Authors also mentioned the need for more holistic policies [51,53,54], with the most common policy referenced being the extension of care beyond age 18 (to age 21 or 25) and better support of youth transitioning from care [48,54,55,56,57,58,59,60,61,62,63]. Other policy suggestions included the need for youth voices in policy decisions [64,65]. Some authors also discussed the need to address systemic disadvantages faced by families involved with child welfare, including poverty, racism, colonialism, and ableism [66,67,68,69,70,71,72,73,74,75,76,77,78,79]. The latter reviews were especially concerned with why certain groups were overrepresented in child welfare, with some review authors detailing systemic bias [74,75,80] and others discussing a higher risk for maltreatment due to a range of preventable societal and community risk factors, such as poor housing and experiences of poverty [71].

#### 3.4.2. Community-Level Suggestions for Improvements

The recommendation referred to most commonly for child welfare improvement was increased multi- and inter-disciplinary collaboration. Specific suggestions for how to do so varied and included: dialogues across key groups of stakeholders, such as caseworkers, children/youth, birth parents, foster parents, kinship carers, residential care workers, medical providers, school personnel, juvenile detention facilities, members from the court system, emergency shelter workers, researchers and government leaders [51,58,81,82,83,84,85,86]; increased information and data sharing [56,81,87,88,89]; cross-disciplinary training [53,90,91]; collaboration during treatment planning and implementation [92]; and increased team communication and feedback loops [93]. Many authors also discussed the need for increased access to services, including more services in the community and reduced barriers to existing services [57,67,87,94,95,96,97,98,99,100].

#### 3.4.3. Institutional-Level Suggestions for Improvements

Review authors acknowledged that certain child welfare populations needed to be served better: ethnically and racially diverse families and children [67,71,72,73,74,75,76,77,78,80,101,102,103,104,105,106,107,108,109,110,111,112]; families experiencing mental health or substance use concerns [53,91,106,113,114,115,116,117,118,119,120,121,122,123,124,125,126,127]; youth transitioning from care [54,59,60,62,63,65,96,128,129,130,131,132,133]; families experiencing low socioeconomic status [66,68,70]; children with complex needs [88,134,135,136,137,138,139]; families experiencing intimate partner violence [140,141,142]; and LGBTQ+ families and children [57,69,143,144]. Other important groups identified by review authors to be served better that were not listed in Table 6 included siblings entering care [145,146], kinship carers [83,97,147], and children with overall health and mental health concerns (the latter was a concern so ubiquitous across included articles that it was not coded as a unique concern).

In addition to these important populations, authors also acknowledged several important principles that should inform services: evidence-based and effective [50,56,57,82,84,88,89,91,92,95,120,148,149,150,151,152,153,154]; tailored to the specific family/child (including development of interventions for the unique needs of children involved with child welfare or in out-of-home care) [53,56,57,59,67,147,151,153,154,155]; culturally sensitive, appropriate, or safe [54,59,67,84,89,97,116,120,149,156,157]; preventative approach [56,98,151,153]; developmentally sensitive and age-appropriate [54,57,97,99,120,150]; trauma-informed [64,82,120,156]; comprehensive [57,62,82,88,93,116,148]; and strengths-based [120,158].

Many reviews discussed the need for robust child welfare research, such as use of randomized controlled trials, quasi-experimental designs, longitudinal research, and other recommendations for ‘rigorous’ research [50,52,62,86,91,97,99,129,130,155,158,159,160,161,162,163,164]. Many reviews also discussed the need to include the voices of those being served by child welfare, including biological family members, foster parents and kinship carers, and especially the voices of children involved in child welfare or in out-of-home care [52,56,62,64,85,89,91,93,97,162,165,166,167].

#### 3.4.4. Relationship-Level Suggestions for Improvements 

There were numerous references to the importance of collaborative relationships between healthcare and social service providers and families and children involved with child welfare. There were a variety of roles expected of providers, such as surveillance [49,92,96,99,168,169], collaboration with other providers and coordination of services [91,93,170], and documentation [56,82,88,99,168,170]. However, four specific relational roles were discussed in many of the articles (see Table 6). These roles included the importance of comprehensive assessments for children involved in child welfare or in out-of-home care [50,52,56,58,82,83,84,91,93,96,99,130,134,148,153,159,160,169,171,172,173,174], advocacy on behalf of children and families [82,96,134,168,170,174], referral to effective or evidence-based services [50,54,55,168,172,175], and a variety of ways that children and families could be supported [49,50,55,56,58,61,82,83,87,88,91,96,120,130,145,152,153,168,171,173,174,175,176,177], such as through psychoeducation, enhancing support networks, or through help navigating or overcoming barriers in the system.

#### 3.4.5. Individual-Level Suggestions for Improvements 

At the individual level, the proposal most frequently identified by authors for child welfare improvement involved training of healthcare and social service professionals, including child welfare professionals, foster carers, and kinship carers. Training suggestions most often focused on the awareness and ability to respond to the unique needs of children and families involved in child welfare or out-of-home care [54,83,88,89,93,116,130,134,156,159,168,178,179]; training on health topics [48,56,57,153,178]; and training on how to navigate child welfare and out-of-home care and by extension how to support children and families who are navigating these systems [88,180].

## 4. Discussion

This scoping review sought to summarize reviews addressing children, youth, and families coming into contact with child welfare in high-income countries. A particular focus was given to authors’ recommendations for improving child welfare across socioecological levels. 

Over 20 years ago, Waldfogel [181] identified five main issues with the existing CPS system in the United States:

1. over-inclusion (for example, unnecessary child protection reports);
2. under-inclusion (such as, inadequate support and protection for vulnerable children, particularly in response to sexual abuse and exploitation);
3. service capacity (for example, severe funding shortages; gaps in services);
4. service delivery (such as, adversarial relationships with parents; discrimination towards low-income families); and
5. service orientation (for example, residual child welfare, or only providing support as a last resort when all other avenues fail; crisis intervention orientated).

Many of these issues were raised by authors of included reviews as concerns that continue to affect child welfare, including the over-representation of certain populations (e.g., Indigenous families, Black families, and families facing poverty), the under-inclusion of certain populations (e.g., children with disabilities experiencing maltreatment), service capacity concerns (e.g., underfunding and lack of political support for child welfare, difficulty in accessing services in communities), service delivery concerns (e.g., bias in professionals towards racialized, impoverished families), and service orientation concerns (e.g., a tendency towards reactive, additive protocols, priorities, and requirements in child welfare). A fundamental question to ask, then, is: Where to start with improving child welfare for children, families, and for the professionals whose role it is support them? 

Below we organize our discussion by reform strategies across socioecological levels. Many of these themes correspond with policy and practice recommendations issued by a Lancet Commission regarding institutionalisation and deinstitutionalisation of children, including the need to improve children’s outcomes and key elements of a national reform system [182]. Specific subthemes, such as the need for multi- and inter-disciplinary collaboration, a revaluation of workforce involvement and better evaluation of child outcomes, also correspond with reviews on public health approaches to child welfare [13,22] and a recent policy review on foster care [183]. 

### 4.1. Societal-Level Reform 

A key concern for several review authors was the need for holistic policy reform. Authors [13] noted that there was “no ideal one-size-fits-all reform process” as “a variety of informed strategies to promote child, family and community health, well-being and social care are required across multiple services and programs, each with their own particular organizational and community context” (p. 5). 

An important concern for a smaller subset of articles was systemic discrimination—including racism, colonialism, ableism, and poverty—and how these factors put some families at increased risk of contact with child welfare. This is in comparison to most articles that focused on more ‘malleable’ factors such as individual factors (e.g., training), institutional factors (e.g., principles of good services), and community factors (e.g., need for increased collaboration) (discussed further below). As such, there tended to be a theoretical division in the included reviews regarding where improvement in child welfare would be most beneficial. Some authors focused on factors at the macro level, which are more difficult to shift (e.g., poverty) but would benefit a significant number of people, thus potentially reducing the need for child welfare involvement (primary prevention). Other authors focused on factors at the practice level; these factors can be more malleable (e.g., parenting skills) in the short- and medium-term to address the maltreatment the child is experiencing or had experienced in the past (secondary and tertiary prevention). Policy and decision-makers conducting child welfare reform must address how to meaningfully engage these two distinct groups who both have commitment and important ideas for improving child welfare. 

### 4.2. Community-Level Reform 

The most common proposal to improve child welfare identified by authors was the need for improved multi- and inter-disciplinary collaboration and coordination of services. While specific suggestions for improving collaboration and coordination across disciplines were sometimes offered [13,184,185], this theme was often not well described by authors. 

An important proposal from several reviews was that the protection of children should involve more than just child welfare workers [13,22] and one review [13] noted that child welfare reform ideally required a “re-imagining of which workforce(s) are in scope, and re-crafting of the workforce knowledge, skills and values to ensure system capacity and practitioner capability to deliver properly targeted prevention strategies” (p. 8). Sectors that are in scope at different points of the child’s life include education, healthcare, social services, housing, and justice [13,22]. 

### 4.3. Institutional-Level Reform 

At the institutional level, review authors identified that there were a number of key populations who need to be served better in child welfare; they also consistently referenced a number of fundamental principles to effectively serve these populations. A key concern for authors was how do so with limited resources and systemic barriers. For example, Font et al. [183] discussed how foster care reform efforts to date have been mostly “additive, layering more protocols, priorities, and requirements to foster care practice over time” (p. 19). One strategy to better serve populations referenced by several included reviews was the need to understand the components of services, interventions, and programming [94,186], and to effectively evaluate them, in order to improve their effectiveness. For example, improving foster care so it addresses children’s well-being and safety (vs. process indicators, such as number of placements) requires (a) assessing what outcome measures accurately determine child safety and well-being, (b) discarding measures that are not clearly associated with child well-being and safety, and (c) evaluating programs and services with these measures, in order to determine which components are essential to child outcomes of safety and well-being [183]. It also ideally requires the use of measures that are not easily manipulated by overworked child welfare workers and administrators, such as data from health care, juvenile justice, and education (e.g., student honour role, sickness days, emergency room visits) [183]. This point resonates well with reviews that emphasized the need for integrative data systems [13,22]. 

A review by Herbert et al. [187] discussing components of child welfare services presented the collective program logic of multidisciplinary teams responding to child physical and sexual abuse. In delineating the assumptions of each program component, the authors [187] strived to move towards ‘opening the black box’ of program theory so that evidence-bases can be developed “not around programs or models, but around the common components that appear across many programs” (p. 9). These authors [187] argued that such work is necessary to evaluate the “explanation underlying the connection between team and centre activities and their intended outcomes” (p. 10). For example, does a “warm referral” (a facilitated introduction to another service provider) to therapeutic services increase engagement and completion of services offered by multidisciplinary teams, as well as improve child or family outcomes, as is assumed?

Aside from better evaluation of components of services, programs, and interventions, review authors also commented on the need for services to be tailored to and representative of those using the services. This especially involved an investment in culturally appropriate services and effectiveness research that accounts for diversity in racial, ethnic and cultural groups [67,77].

### 4.4. Relationship-Level Reform 

Many of the reviews summarized within this scoping review discussed the importance of relational aspects of service provision, including comprehensive assessment, advocacy, referral, and support. More specifically, many authors noted that children involved in child welfare need comprehensive initial and ongoing assessment that includes, but is broader than mental health symptoms [187,188,189], as children may need referral to services and support before serious problems develop, including assessment and referral or support for their social-emotional well-being [148,188]; physical health [190], including developmental needs [99], dental care [191], sexual health [172], substance use or prenatal substance exposure [124]; and food-related behaviours [192]. Assessments of needs were recommended for various service entry and exit times, including entry into care, at regular intervals while in care [97], as well as exits from care [62] and transitions to adult mental health services [96]. Assessments of safety of children involved in child welfare were also of concern to review authors [193,194,195]. With respect to advocacy, review authors argued that healthcare and social service providers, including child welfare workers, should advocate for services, supports, and opportunities to increase the well-being and participation of children and families throughout and beyond their involvement with child welfare [96,134,196,197,198]. As was noted above, support was broadly conceived by review authors to include a variety of activities to improve the lives and well-being of children and families involved in child welfare, including psychoeducation, enhancing support networks, and help to navigate the child welfare system, among others. Often, review authors simply noted that health and social services providers should “support” children and families. 

In spite of significant theoretical differences informing child welfare research and summaries in this review, review authors [199,200] noted that one area of overlap is the importance of forming relationships during service provision. In social services there is an increasing focus on the need for services to be relationship-based [201]; however, in social services practice, including child welfare practice, there continues to be an emphasis on administrative processes, such as documentation, rather than relational processes [202]. Authors [203] have summarized a number of occupational risk factors faced by child welfare workers, such as “a personal history of maltreatment, inadequate support, high workloads, low salaries, long working hours, exposure to clients’ trauma … violence and aggression from clients…political interference…and schedules which negatively impacted services rendered to clients” (p. 7). These risk factors and other factors across socioecological levels (e.g., demographic characteristics of child welfare workers; organizational support for workers, including peer support and supportive supervision) [204,205,206,207] lead to high staff turnover, which negatively impacts relationships with children and families. One of the key policy strategies raised by authors to reduce the occupational risk factors of child welfare workers was increased governmental funding for more workers, in order to reduce caseloads. 

### 4.5. Individual-Level Reform 

Blome et al. [208] argued that focusing on funding to reduce caseloads is important but noted that caseworkers still need to be skilled: “focusing on size may obfuscate other larger issues within the agency like the level of education and experience required for frontline positions” [203]. This scoping review made clear the—arguably impossible—variety of administrative and clinical skills that child welfare workers were expected to have. Authors from different disciplines (education, healthcare, child welfare, criminal justice) all concluded by suggesting how better training of child welfare workers was needed–very few authors concluded that their own discipline needed to better support children experiencing maltreatment. While many reviews recommended better training for child welfare professionals, very few reviews evaluated training strategies for child welfare professionals [142,209,210]. 

The unrealistic expectations towards child welfare workers by society underscores the need for a “comprehensive workforce needs analysis in collaboration with all relevant stakeholders (within universal, secondary and tertiary services), followed by development of a plan to address current and ongoing system and service user needs” [13]. As has been stated by several review authors (and stated several times in the present review), the important needs of children experiencing maltreatment cannot be served by child welfare alone—an investment of the skills and resources from many sectors is required.

### 4.6. Limitations, Future Research, and Implications 

As this scoping review summarized published reviews in between 2010 and 2021, it is limited by the content of available reviews and excludes all articles that have not yet been included in published reviews. In our attempt to succinctly summarize the wide variety of author-proposed suggestions for child welfare reform, we have not critically appraised individual reviews or their empirical research. For example, a number of reviews spoke to the over-inclusion of families experiencing poverty in child welfare due to factors like implicit bias, but we have not critically examined individual studies that have sought to address if low-income families are referred to child welfare because of implicit bias. A recent commentary by Barth and colleagues [211] is an example of an accessible summary of individual studies seeking to address pressing child welfare reform queries. Given the sheer volume of reviews summarized, we have also not analyzed or assessed individual programs, services, or policy recommendations. For example, several reviews summarized specific child welfare services, including evidence-based programs to support children and families. We have not critically appraised these reviews or their included studies and as such were not able to make recommendations about the value of specific programs and services. 

The 433 reviews summarized in this scoping review offer a variety of informed strategies to promote child, family and community well-being that have important and wide-ranging practice implications. Thematic analysis suggests that many of the issues plaguing child welfare have not changed in the past few decades. This suggests that while much evidence-based research has accumulated over the past decade in terms of services and supports for children and families involved in child welfare, as in other areas of violence prevention, the leadership, governance, and political will necessary to tackle child welfare reform is underdeveloped [212]. As such, future research and practice in the area of child welfare reform needs to move beyond identifying what should be done and examine how to undertake reform efforts with accompanying evaluation. For example, many reviews over the past decade have suggested that better collaboration between child welfare and other sectors is needed (the “what”), but it is less clear how this collaboration should be undertaken. In line with how collaboration might be undertaken, Luckock et al. [199] have summarized innovative models of practice at the interface between the United Kingdom’s National Health Service and child and family social work. One relevant example of multi-sector collaboration cited by the authors [199] included “approaches to multi-agency team working in children’s services [that] have emerged in which ‘co-location’ of health, social and other practitioners (especially police) is the preferred means of service integration at those points where ‘early help’ needs better alignment with a ‘child protection’ response” (p. 65). While the authors [199] noted that “developments of this kind are becoming widely established across England” (p. 65), their review found only two evaluations of these efforts, neither of which accounted for child and family well-being outcomes. As such, like Luckock et al. [199], future work should detail how child welfare reform can be meaningfully executed and these efforts should evaluate if and how such innovations improve the lives of children and families.

Future research is needed to assess the readiness of child welfare for any specific avenue of reform, including the readiness to evaluate its effectiveness at the level of child and family well-being. Such assessment, especially for widespread reform, can be challenging. For example, given that in Canada it is the responsibility of individual provinces and territories to implement child welfare services, it is politically and practically difficult to implement and assess the effectiveness of any national child welfare reform efforts. Important avenues of child welfare reform in Canada are seen with Indigenous efforts for child welfare sovereignty; however, these efforts are mired by funding and infrastructure (e.g., service delivery) issues [213,214]. Any particular avenue of reform, including the example of multi-sector collaboration discussed above, must therefore be tailored to the specific jurisdiction of interest and any particular contextual challenges.

## 5. Conclusions

This scoping review sought to summarize reviews addressing children, youth, and families coming into contact with child welfare in high-income countries by focusing on authors’ recommendations for improving child welfare across socioecological levels. Several key strategies were discussed in this review, such as holistic policy reform that moves away from layered, additive, reactive protocols, priorities, and requirements; addressing underlying disparities and social determinants of health problems; meaningful engagement with children and youth involved in child welfare at all levels of decision-making; multi- and inter-disciplinary collaboration, including a comprehensive workforce analysis to re-imagine what workforces are in scope; component analysis of all levels of child welfare services and evaluation of services with attention to child well-being and safety outcomes; and increased access to services in communities, including services that follow key principles (e.g., trauma-informed, comprehensive, tailored, relationship-based), accountability and better preventive services for populations that are overrepresented in child welfare (e.g., racialized groups and families experiencing poverty). As several authors noted, there is no one way to conduct a child welfare reform process. An important next step is to formulate what policy solutions are likely to lead to the greatest improvement in safety and well-being for children experiencing maltreatment and to rigorously evaluate these solutions at the level of child and family well-being. 

## Figures and Tables

**Table 1:** Inclusion criteria.

Category	Inclusion Criteria
Population	Children, youth (0–25 years of age) and families with prior or current involvement with child welfare.
Concept	Study author recommendations to improve child welfare at the societal-level (e.g., policy), community-level (e.g., coordination of services), institutional-level (e.g., child welfare initiatives), relationship-level (e.g., social worker skills), and individual-level (e.g., training).
Context	Peer-reviewed reviews that summarized recommendations to improve child welfare in high-income countries. Reviews could include systematic reviews, meta-analyses, qualitative reviews (e.g., meta-syntheses), rapid reviews, scoping reviews, and narrative reviews.
Timeline	2010 to 2021 (when the search was conducted)

**Table 2:** Example of database search strategy.

Example of Database Search Strategy
Database: Ovid MEDLINE(R) ALL <1946 to June 04, 2021> Search Strategy: -------------------------------------------------------------------------------- 1 Child Protective Services/ (602) 2 (child * adj3 (welfare or aid)).tw, kw. (5015) 3 (child * protect * adj3 (service? or agenc * or organi?ation?)).tw, kw. (1560) 4 (“foster care” or “foster home?” or “residential care” or “kin care” or “kinship care”). tw. (6280) 5 (out-of-home adj3 (placement? or care)). tw. (894) 6 or/1-5 (12838) 7 exp child/ or exp infant/ (2552666) 8 (child * or girl or girls or boy or boys or infant * or baby or babies or toddler * or preschool * or pre-school * or “young person” or “young people” or teen * or adolescen * or youth *).tw. (2106256) 9 or/7-8 (3282693) 10 6 and 9 (9547) 11 meta-analysis/ or “systematic review”/ or review/ (2915611) 12 (review? or meta-analy * or metaanaly * or metasynthe * or meta-synthe * or (information adj2 synthesis) or (data adj2 synthesis)).tw. (1971484) 13 ((systematic or state-of-the-art or scoping or literature or umbrella) adj3 (review * or bibliographic * or overview * or assessment *)).tw. (512487) 14 “scoping study”.tw. (339) 15 or/11-14 (3640612) 16 10 and 15 (1374) 17 limit 16 to yr=”2010 -Current” (752)

**Table 3:** Types of Reviews.

	Count ^1^
Narrative reviews	249
Systematic reviews	118
Scoping reviews	31
Meta-analyses	23
Integrative reviews	8
Rapid reviews	5
Meta-syntheses	3
Mapping review	1

^1^ Some reviews were both systematic reviews and meta-analyses, so a sum of the counts does not equal the total number of included articles (*n* = 433).

**Table 4:** Population Focus of Included Articles.

	Count ^1^
Involved with child welfare	128
Families	51
Parents	22
Children	55
Out-of-home care	367
Foster carers	28
Kinship carers	12
Parents	6
Children	321
○ Out-of-home care	57
○ Foster care	141
○ Kinship care	83
○ Residential care	24
○ Adoption	16
Child welfare organizations	57
Child welfare professionals	46
Interdisciplinary focus	22

^1^ Some review articles addressed multiple populations, so a sum of the counts does not equal the total number of included articles (*n* = 433).

**Table 5:** Thematic Focus of Included Articles. ^1^

	Count ^2^
Society—laws and policies	
Cross-country analysis of aspects of child welfare	20
International actors influencing child welfare	15
Human rights	7
National child welfare structure	68
National child welfare policies and legislation	51
Systemic disadvantage in child welfare	47
National institutional actors influencing child welfare	11
Community—relationships among organizations, institutions and informal networks	
Collaboration models, strategies, and components	40
Institution—characteristics and rules for operations	
Child welfare organizational policies, procedures, and overall environment	38
Child welfare workforce	17
Child welfare organizational performance and evaluation	12
Professional support for child welfare professionals	9
**Interventions, services, programs and outcomes associated with different sectors**	
Child welfare	252
○ Placement	94
○ Biological family	64
○ Usage of child welfare services	52
○ Participation in child welfare	48
○ Transition from care	46
○ Safety	42
○ Foster/kinship care (as a service)	41
Health	197
○ Mental health	121
○ Social health	79
○ Physical health	69
○ Usage of health services	22
Education	41
Research	22
Justice	21
Housing	19
Individual—knowledge, attitudes, skills, etc.	
**Child welfare professionals**	
Knowledge, skills, abilities, needs	38
Decision-making	23
Personal characteristics	13
Impact of role	7
**Foster/kinship carers**	
Knowledge, skills, abilities, needs	40
Personal characteristics	15

^1^ In the table the shaded headings indicate a level of the socioecological model [40], which was used to organize the themes. Bolded headings represent higher-order themes that were used to organize relevant sub-themes. The bolded headings are not associated with coded articles in the Excel file. ^2^ Most review articles had multiple themes, so a sum of the counts does not equal the total number of included articles (*n* = 433).

**Table 6:** Authors’ Recommendations for Improving Child Welfare Across Socioecological Levels. ^1^

	Count ^2^
Society—laws and policies	
Political (policy/legislative) support and associated funding for child welfare	56
Need for holistic, not fragmented policies	52
Address systemic disadvantages	46
Youth voices in decision-making and policies	24
Community—relationships among organizations, institutions and informal networks	
Multi- and inter-disciplinary collaboration and coordination	118
Increased access to services	67
Cross-disciplinary training	35
Information sharing	22
Institution—institutional characteristics and rules for operations	
**Cross-disciplinary focus**	
**Important populations to serve better**	
○ Ethnically/racially diverse families and children	59
○ Families experiencing substance use/mental health concerns	43
○ Youth transitioning from care	28
○ Families experiencing low socioeconomic status	20
○ Children with complex needs (such as chronic illness, disability or sensory impairment and needs additional support on a daily basis)	15
○ Families experiencing intimate partner violence	11
○ LGBTQ+ families and children	6
**Important principles to inform services**	
○ Evidence-based/effective	87
○ Tailored (specific to needs of family/child)	64
○ Culturally sensitive/appropriate/safe	58
○ Preventative approach	47
○ Developmentally sensitive/age appropriate	44
○ Trauma-informed	44
○ Comprehensive	37
○ Strengths-based	23
**Child welfare research focus**	
○ Robust research (randomized controlled trials/longitudinal)	155
○ Qualitative research (voices of children, youth, and families)	66
Relationships—formal and informal relationships	
**Improving relationships with families/children or assistance for families/children through:**	
Support	104
Assessment	83
Advocacy	30
Referral	25
Individual—knowledge, attitudes, skills, etc.	
**Training for healthcare, social service providers (including child welfare professionals), and foster carers**	
Training on unique needs of children/families involved in child welfare or out-of-home care	108
Training on health topics	18
Training on navigating child welfare and out-of-home care	12

^1^ In the table the shaded rows indicate a level of the socioecological model [40], which was used to organize the themes. Bolded headings represent higher-order themes that were used to organize relevant sub-themes. The bolded headings are not associated with coded articles in the Excel file. ^2^ Most review articles had multiple suggestions for child welfare reform, so a sum of the counts does not equal the total number of included articles (*n* = 433).

## Data Availability

The Excel file with all of the coding is available on Dryad Data Repository (https://datadryad.org/stash, doi:10.5061/dryad.jsxksn0dc). All other material and data used for this review are available in the paper and tables.
